# The other side of COVID-19: A cross-sectional study on mental health in a sample of Italian nurses during the second wave

**DOI:** 10.3389/fpsyt.2023.1083693

**Published:** 2023-03-01

**Authors:** Erika Renzi, Valentin Imeshtari, Dima Masud, Valentina Baccolini, Giuseppe Migliara, Giulia Gasperini, Corrado De Vito, Carolina Marzuillo, Paolo Villari, Azzurra Massimi

**Affiliations:** ^1^Department of Public Health and Infectious Diseases, Sapienza University of Rome, Rome, Italy; ^2^Emergency Department, Sandro Pertini Hospital, Rome, Italy; ^3^Department of Translational and Precision Medicine, Umberto I Teaching Hospital, Rome, Italy; ^4^Department of Biomedicine and Prevention, University of Rome Tor Vergata, Rome, Italy

**Keywords:** nursing, mental health, COVID-19, post-traumatic stress disorder, generalized anxiety disorder

## Abstract

**Introduction:**

The COVID-19 pandemic has led to a drastic increase in the workload of healthcare professionals, particularly nurses, with serious consequences for their psychological well-being. Our study aimed to identify demographic and work-related factors, as well as clinical predictors of post-traumatic stress disorder (PTSD) and generalized anxiety disorder (GAD), in nurses employed during the COVID-19 pandemic.

**Methods:**

We carried out a cross-sectional study between December 2020 and April 2021 on nurses employed during the COVID-19 second wave (October - December 2020). We evaluated PTSD and GAD using two validated questionnaires: i) the Impact of Event Scale – Revised (IES-R); and ii) General Anxiety Disorder –7 (GAD-7).

**Results:**

Overall, 400 nurses, whose mean age was 34.3 years (SD ± 11.7), were included in the study. Most were female (78.5%), unmarried (58.5%) and employed in the central (61.5%) regions of Italy. A total of 56.8% of all participants had clinical predictors of PTSD, recording a median IES-R score (IQR) of 37.0 (22.0, 51.0) (range 1-84; cut-off >33 for PTSD). Furthermore, 50% of respondents reported moderate-to-severe symptoms consistent with GAD, recording a median GAD-7 score (IQR) of 9.5 (6.0,14.0) (range 0-21; cut-off >10 for GAD). Multivariable analysis showed that moderate-to-severe GAD (aOR = 4.54, 95% CI: 2.93 - 7.05), being employed in the critical care area (aOR = 1.74, 95% CI: 1.01 - 3.00) and being female (aOR= 1.88, 95% CI: 1.09 - 3.22) were significantly associated with the presence of clinical predictors of PTSD.

**Discussion:**

The levels of PTSD symptoms and anxiety among nurses were high during the pandemic. PTSD and GAD represent a public health problem that should be addressed in the post-pandemic period. Healthcare organizations need to activate specific support and rehabilitation networks and programs for healthcare professionals employed during the COVID-19 pandemic.

## 1. Introduction

Since the beginning of the COVID-19 pandemic, healthcare workers (HCWs) were in the front line, assisting patients with COVID-19; they faced an unexpected and sudden increase in healthcare demands and were exposed to a high risk of contracting the disease (1). Furthermore, because of the fear of contagion and the social-distance measures put in place to contain the pandemic, HCWs often could not or chose not to see their families and friends for long periods, so they found themselves alone and with no emotional support. Such conditions are known to have negative effects on the psychological health of HCWs (2, 3).

Psychological sequelae secondary to the outbreak of an epidemic have already been reported for Middle East respiratory syndrome (MERS) in 2012 and severe acute respiratory syndrome (SARS) in 2003, in which symptoms of post-traumatic stress disorder (PTSD), sleep disorders, social isolation, work-related stress, burnout, and generalized anxiety disorder (GAD) were reported in HCWs (4–7). In the case of the COVID-19 pandemic, the scientific literature shows that HCWs, especially nurses and women who worked in the front line and in emergency areas, experienced more severe mental health symptoms than others (3). According to a meta-review of systematic reviews, GAD and PTSD were the most prevalent COVID-19 pandemic-related mental health conditions affecting HCWs, especially nurses (8). Several more detailed studies, conducted after the first wave of the pandemic in China, reported that HCWs suffered from anxiety and stress-related symptoms, with prevalence ranging from 28.5 to 36.1% and 24 to 73.4%, respectively (9–11). In addition, some studies have reported high levels of anxiety and acute stress disorders in nurses more than a year after the start of the pandemic (12, 13).

Focusing attention on the psychological impact of COVID-19 on HCWs, particularly on the Italian nurses who were among the first in Europe to deal with the pandemic and to assist COVID-19 patients (14), is crucial to investigate if such HCWs are to be offered the right tools to face this extraordinary scenario and to preserve their mental health, both as individuals and as actors in the National Health Service. In the Italian context, relatively few studies have investigated the prevalence of GAD and PTSD in HCWs, and most of these were conducted exclusively during the first wave of COVID-19 (15, 16), focusing on local contexts (12, 17, 18). Given this, we conducted a study on a national sample of nurses employed during the second wave of the COVID-19 pandemic to investigate the long-term impact on nurses’ mental health, with the specific aims of (i) determining the prevalence of symptoms of potential GAD and PTSD; (ii) identifying possible predictors of PTSD.

## 2. Materials and methods

### 2.1. Setting and participants

We conducted a cross-sectional survey, using a convenience sample of Italian nurses who worked on the front line during the second wave of the COVID-19 pandemic (October–December 2020) (19, 20). The survey followed the Checklist for Reporting Results of Internet E-Surveys (CHERRIES) (21) (Supplementary Material S1) and was disseminated through social-media platforms (Facebook, Telegram, Instagram), as well as specific social-media groups for nurses. Data collection was performed between December 2020 and April 2021. Nurses were invited to take part voluntarily in an online survey accessible *via* smartphone through a Google Form link. The study was performed in accordance with the World Medical Association Declaration of Helsinki. Participants were asked for their consent and were guaranteed anonymity in the information collected. The institutional ethics board of the Umberto I Teaching Hospital/Sapienza University of Rome approved this study (protocol 0489/2021).

### 2.2. Questionnaire

We developed a 41-item survey based on the literature. The questionnaire comprised three sections. The first section included 12 items that aimed to collect socio-demographic and occupational data: gender, age, nationality, marital status, employment information (department, area, employment in COVID-19 area during the first wave, and geographical context), and COVID-19 personal history (previous SARS-CoV-2 infection and experience). The second section aimed to investigate anxiety disorders: in this case, Generalized Anxiety Disorder-7 (GAD-7), a questionnaire for GAD screening, was used in its validated Italian version. The scale assigns a score from 0 (not at all) to 3 (almost every day) based on the frequency of reported symptoms over the previous 14 days. The total GAD-7 score for the seven items ranges from 0 to 21. Based on the total score, it is possible to stratify the presence of symptoms of GAD (0–4: minimal anxiety; 5–9: mild anxiety; 10–14: moderate anxiety; 15–21: severe anxiety) (22). The third section assessed acute stress reactions and the increased likelihood of having PTSD using a validated questionnaire, Impact of Event Scale-Revised (IES-R), which has demonstrated good internal consistency in the Italian version (23). IES-R is a 22-item scale that is rated from 0 (not at all) to 4 (extremely) with respect to how distressing each item was during the last 7 days. For the purposes of our study, we modified the IES-R instructions by contextualizing the questionnaire to the COVID-19 experience of the 7 days prior. The total IES-R score for the 22 items ranges from 0 to 88. The scale scores are determined from three subscales, which reflect intrusion (8 items), avoidance (8 items), and hyperarousal (6 items; see Table 1 for definitions).

**Table 1:** Impact of Event Scale-Revised (IES-R) subscale definition.

**IES-R subscale (PTSD symptoms)**	**Definition**
*Avoidance*	The practice or an instance of keeping away from particular situations, environments, individuals, or things because of either (a) the anticipated negative consequence of such an encounter or (b) anxious or painful feelings associated with them (APA Dictionary). *For example: avoiding reminders of trauma; losing interest in activities*
*Hyperarousal*	One of three sets of criteria used to diagnose post-traumatic stress disorder and acute stress disorder. Symptoms of hyperarousal include exaggerated startle response, disturbed sleep, difficulty in concentrating or remembering, and excessive vigilance (APA Dictionary). *For example: difficulty sleeping; angry outbursts*
*Intrusion (or intrusive thoughts)*	Mental events that interrupt the flow of task-related thoughts in spite of efforts to avoid them (APA Dictionary). *For example: nightmares; reliving trauma*

IES-R, impact of Event Scale-Revised; PTSD, post-traumatic stress disorder.

### 2.3. Statistical analysis

Descriptive statistics were obtained using the median and interquartile range, or mean and standard deviation, for continuous variables, and proportions for dichotomous and categorical variables. The COVID-19 employment area was categorized into five categories: critical care (i.e., emergency departments, intensive care units), medical wards (also including nursing homes), surgical wards, primary care (i.e., COVID-19 vaccination services, COVID-19 testing programs, contact tracing units, and home-care services for COVID-19 patients), and other (i.e., mental-health units, rehabilitation units). The age of the nurses was classified according to the average age (35 years). The presence or absence of symptoms of PTSD and GAD were assessed using validated questionnaires and were classified as either being present or absent (two variables). To assess GAD-7, we classified a score > 10 as “presence of moderate/severe symptoms of GAD”. PTSD was classified into two levels: IES-R scores ≥33 were classified as “presence of symptoms of PTSD” and scores <33 as “absence of symptoms of PTSD”. For the univariable analysis, Pearson’s chi-squared test was used for dichotomous and categorical variables, while the Mann–Whitney U Test was used to compare continuous variables between females and males. A multivariable logistic-regression model was built to identify predictors of PTSD. According to the Hosmer and Lemeshow logistic-regression model building strategy, the variables examined by univariate analysis using the appropriate statistical test were included in the model when the *p*-value was less than 0.25 (see Supplementary Material S2). We also included variables described by opinion of experts and literature to provide a complete control of confounding. All variables initially tested were retained in the final model because their exclusion altered the aORs of the other variables. The only exception was the variable “years of working” which was removed due to collinearity with the variable “age”. The final model consisted of the following variables: sex (female vs. male), age (≤ 35 and > 35 years), marital status (married/engaged vs. single/unmarried/separated/divorced), region of employment (northern Italy vs. center, south Italy, and Islands), nurses employed in COVID-19 wards during the first and second waves, COVID-19 employment wards/area (primary care, critical care, medical wards, surgical wards and other), COVID-19 previous infection, symptomatic COVID-19 positive member of family/friends and presence of moderate-to-severe GAD (GAD-7 score ≥ 10). Results were expressed as adjusted odds ratios (aOR), their 95% confidence intervals (CI), and *p*-value. A *p*-value <0.05 was considered statistically significant. All analyses were performed using Stata (StataCorp LLC, 4905 Lakeway Drive, College Station, TX, United States), version 17.0.

## 3. Results

### 3.1. Characteristics of the sample

A total of 400 nurses employed in 19 of the 21 Italian Regions (19 administrative Regions and two autonomous provinces) during the second wave of the COVID-19 pandemic completed the survey. In line with the gender distribution of nurses in Italy [76.45% women (24)], most of the sample was female (78.7%), unmarried (58.5%), and employed in central Italy (61.5%), with a mean age of 34.3 ± 11.8 years. The years of professional experience for the overall sample ranged from 0 to 43 years (mean = 16 years, SD= ± 11.9). Most respondents worked in COVID-19 units during both the first and second waves of the pandemic (195; 51.3%); the main areas of work were critical (37.0%) and primary (35.3%) care. Finally, 19% of the sample reported a previous SARS-CoV-2 infection (Table 2).

**Table 2:** General characteristics of the survey sample.

**Variable**	**Frequency (%)**	**Mean (±SD)**
Age		34.3 ± 11.8
Sex		
Female	315 (78.7)	
Male	85 (21.3)	
Years of working		16.1 ± 11.9
≤1 year	107 (26.8)	
2–5 years	121 (30.2)	
5–10 years	45 (11.3)	
>10 years	125 (31.2)	
Missing system	2 (0.5)	
Marital status		
Single/unmarried	214 (53.5)	
Married/engaged	166 (41.5)	
Separated/divorced	20 (5.0)	
Region of employment		
North	116 (29.0)	
Centre	246 (61.5)	
South and Island	38 (9.5)	
Employed in COVID-19 wards during first wave (March–May 2020)		
Yes	195 (51.3)	
No	205 (48.7)	
COVID-19 employment wards/area		
Critical care	148 (37.0)	
Medical wards	89 (22.2)	
Surgical wards	6 (1.5)	
Primary care	141 (35.3)	
Other	16 (4.0)	
COVID-19-positive		
Yes	76 (19.0)	
No	324 (81.0)	
COVID-19 cases among family members/friends		
Yes	83 (20.8)	
No	317 (79.2)	

SD, standard deviation.

### 3.2. Generalized anxiety disorder

The median GAD-7 score (IQR) was 9.5 (6.0,14.0; range: 0–21). Of the four severity categories, only 63 nurses (15.8%) reported as being in a normal state, while the remaining 337 (84.2%) experienced symptoms of anxiety, from mild (*n* = 137, 34.2%) to moderate (*n* = 106, 26.5%), and up to severe GAD (*n* = 94, 23.5%). Based on the cut-off value of 10, the prevalence of symptoms of GAD was 50% (*n* = 200). There was a gender difference in score distribution, with females having a higher median than males: 10.0 (6.0,14.0) vs. 8.0 (6.0,14.0; Table 3).

**Table 3:** Distribution of generalized anxiety disorder and post-traumatic stress disorder scores in the study sample and by gender.

**Severity category (score range)**	***N* (%)**	**Female**	**Male**	***p*-Value**
		***N* (%)**	***N* (%)**	
GAD-7				0.149
Total sample	400 (100)	315 (100)	85 (100)	
Normal (0–4)	63 (15.8)	51 (16.2)	12 (14.1)	
Mild (5–9)	137 (34.2)	99 (31.4)	38 (44.7)	
Moderate (10–14)	106 (26.5)	87 (27.6)	19 (22.4)	
Severe (15–21)	94 (23.5)	78 (24.8)	16 (18.8)	
IES-R				
Total sample	400 (100)	315 (100)	85 (100)	0.012
Absence of symptoms for PTSD (0–32)	173 (44.2)	126 (40.0)	47 (55.3)	
Presence of symptoms for PTSD (33–88)	227 (56.8)	189 (60.0)	38 (44.7)	

GAD-7, generalized anxiety disorder-7 questionnaire; IES-R, impact of Event Scale-Revised; PTSD, post-traumatic stress disorder.

### 3.3. Post-traumatic stress disorder

Participants recorded IES-R scores ranging from 0 to 84, with a median score (IQR) of 37.0 (22.0, 51.0). According to the predefined cut-off for assessing the presence of PTSD symptoms (IES-R scores ≥33), of the 227 participants (56.8%) who scored above the cut-off, 195 (85.9%) attained scores higher than 39 (Table 4). The median IES-R score (IQR) of female nurses was higher than that of male nurses: 38.0 (24.0, 52.0) vs. 30.0 (22.0, 45.0; Table 2). Similar gender differences were found for the median subscale scores (IQR): female nurses had a higher median score (IQR) for the *hyperarousal subscale* [8.0 (4.0, 12.0) vs. 6.0 (3.0, 11.0), *p*-value = 0.034] and *intrusion subscale* [16.0 (9.0, 21.0) vs. 13.0 (6.0, 9.0), *p*-value = 0.007]. The items with the highest median score were those in the *intrusion subscale* (Table 5).

**Table 4:** Median of generalized anxiety disorder and post-traumatic stress disorder scores in the study sample and by gender.

**Scale**	**Total score, median**	**IQR**	**Female**		**Male**		***p*-Value**
			**Total score, median**	**IQR**	**Total score, median**	**IQR**	
GAD-7	9.5	6.0–14.0	10.0	6.0–14.0	8.0	6.0–14.0	0.133
IES-R	37.0	22.0–51.0	38.0	24.0–52.0	30.0	22.0–45.0	0.059

IQR, interquartile range; GAD-7, generalized anxiety disorder-7 questionnaire; IES-R, impact of Event Scale-Revised.

**Table 5:** IES-R subscale scores in the study sample and by gender.

**Impact of Event Scale-Revised (IES-R)**					
**Subscale**	**Items**	**Median (IQR)**			***p*-Value**
		**Total**	**Female**	**Male**	
*Avoidance*					
	I avoided letting myself get upset when I thought about it or was reminded of it	2.0 (1.0, 3.0)			
	I felt as if it had not happened or wasn’t real	1.0 (0.0, 2.0)			
	I stayed away from reminders of it	1.0 (0.0, 2.0)			
	I tried not to think about it	2.0 (1.0, 3.0)			
	I was aware that I still had a lot of feelings about it, but I did not deal with them	2.0 (1.0, 3.0)			
	My feelings about it were kind of numb	1.0 (0.0, 2.0)			
	I tried to remove it from my memory	1.0 (0.0, 2.0)			
	I tried not to talk about it	1.0 (0.0, 2.0)			
	Median score for *Avoidance Subscale*	14.0 (9.0, 19.0)	14.0 (9.0, 19.0)	13.0 (8.0, 18.0)	0.426
*Hyperarousal*					
	I felt irritable and angry	2.0 (1.0, 3.0)			
	I was jumpy and easily startled	2.0 (1.0, 3.0)			
	I had trouble falling asleep.	2.0 (0.0, 3.0)			
	I had trouble concentrating	1.0 (0.0, 2.0)			
	Reminders of it caused me to have physical reactions, such as sweating, trouble breathing, nausea, or a pounding heart	0.0 (0.0, 1.0)			
	I felt watchful and on-guard	2.0 (1.0, 3.0)			
	Median score for *Hyperarousal Subscale*	8.0 (4.0, 11.00)	8.0 (4.0, 12.0)	6.0 (3.0, 11.00)	0.034
*Intrusion*					
	Any reminder brought back feelings about it	2.0 (1.0, 3.0)			
	I had trouble staying asleep	2.0 (0.0, 3.0)			
	Other things kept making me think about it	2.0 (1.0, 3.0)			
	I thought about it when I did not mean to	2.0 (1.0, 3.0)			
	Pictures about it popped into my mind	2.0 (1.0, 3.0)			
	I found myself acting or feeling like I was back at that time	2.0 (1.0, 3.0)			
	I had waves of strong feelings about it	1.0 (0.0, 2.0)			
	I had dreams about it	2.0 (1.0, 3.0)			
	Median score for *Intrusion Subscale*	15.0 (8.0, 20.0)	16.0 (9.0, 21.0)	13.0 (6.0, 19.0)	0.007

*Item response anchors are 0, not at all; 1, a little bit; 2, moderately; 3, quite a bit; 4, extremely; IQR, interquartile range.

### 3.4. Multivariable analysis

At the multivariable analysis, higher odds of PTSD symptoms were found for nurses with presence of symptoms for moderate-to-severe GAD (aOR = 4.54, 95% CI: 2.93 to 7.05). Similarly, being female (aOR= 1.88, 95% CI: 1.09 to 3.22) and employment in the critical care area (aOR = 1.74, 95% CI: 1.01 to 3.00) were associated with the presence of symptoms of PTSD. In contrast, age, marital status, employment in COVID-19 units continually during both the first and second waves, employment in a COVID-19 area in Northern Italy, and previous SARS-CoV-2 infection did not appear to increase the likelihood of having symptoms of PTSD (Table 6).

**Table 6:** Factors associated with post-traumatic stress disorder (IES-R≥33) in nurses.

**Post-traumatic stress disorder**		**aOR**	**95% CI**	***p*-value**
Sex (female)		1.88	1.09–3.22	0.022
Age (≤35 years old)		1.14	0.70–1.85	0.606
Marital status (married)		1.13	0.70–1.84	0.608
Employed in Northern Italy		0.90	0.77–2.10	0.342
Employed in COVID-19 departments during the first and second waves		1.09	0.56–1.45	0.669
COVID-19 employment wards/area				
	Primary care	Ref.		
	Critical care	1.74	1.01–3.00	0.046
	Medical wards	1.33	0.73–2.43	0.353
	Surgical wards	1.12	0.19–6.63	0.901
	Other	1.30	0.41–4.13	0.652
COVID-19-positive		1.48	0.82–2.68	0.197
Symptomatic COVID-19-positive family members/friends		0.81	0.51–1.28	0.362
Generalized anxiety disorder (moderate–severe)		4.54	2.93–7.05	0.001

aOR, odds ratio; CI, confidence interval.

## 4. Discussion and conclusion

This study aimed to assess the prevalence of symptoms of GAD and PTSD among nurses employed during the second wave of COVID-19 in Italy and to evaluate the factors influencing PTSD in this sample.

Overall, the results of the present study confirm that the COVID-19 pandemic has had a profoundly negative effect on the mental health of Italian nurses.

Generalized anxiety disorder symptoms were reported by half the nursing population sample employed during the second wave of the pandemic. The prevalence of symptoms of anxiety appears in line with the international context. In fact, during the global pandemic, anxiety was the most prevalent mental disorder in HCWs and prevalence was significantly higher than in the general population, with a similar distribution in the pooled prevalence across all geographic areas: global 42%, (25) Africa 49% (26), South America 35% (27), Eastern Europe 30% (28), and Southeast Asia 23% (9). These results are also consistent with other studies conducted in Italy and other European countries (25, 29–31). Significant differences were observed in the occurrence and severity of the condition according to gender, with female nurses reporting a higher median GAD-7 score. However, even before the pandemic, anxiety overload in the nursing profession, together with its symptomatic manifestations and their potential effect on patient safety, appeared to be a well-established phenomenon: pre-pandemic studies reported a prevalence of GAD greater than 35% in the nursing population, with a particular impact on female nurses (32–36). Therefore, to rebuild a healthcare system challenged by COVID-19, it will be necessary to increase efforts to screen and diagnose anxiety disorders in healthcare providers.

In the case of PTSD, the recorded IES-R scores suggest the presence of PTSD symptoms in almost 60% of the sample. This is markedly higher than the prevalence shown in pre-pandemic studies, which reported PTSD in 7–21% of nurses (37, 38). However, our findings are still consistent with studies carried out during other emergencies/crisis periods when HCWs, particularly nurses and frontline workers, experienced higher levels of psychological distress, anxiety, and PTSD (39). In addition, recent systematic reviews have reported a prevalence of PTSD among HCWs ranging from 21.5% to 73.5% (25, 40); in studies conducted specifically in Italy, during the current pandemic, the reported levels of PTSD symptoms ranged from 37.2% to 52.6% (16, 31). Another finding that emerged from our study was the gender difference in the distribution of mental-health conditions, with a higher prevalence of psychological symptoms of PTSD in females, which is also in line with a recent systematic review of the literature (41). Women are more likely than men to suffer from psychological disorders due to a combination of multiple biological, social, and gender-role factors. Gender differences are extensively described in the literature and in psychopathology texts: the Diagnostic and Statistical Manual of Mental Disorders (DSM-5) and the U.S. National Center for PTSD define being female as a risk factor for the development of PTSD (42–44). This difference is evident both in the median IES-R score of the sample (female nurses 38.0 vs. male nurses 30.0) and in terms of symptomatology, with a significant difference in the presence of symptoms related to intrusion and hyperarousal. With reference to intrusion symptoms, higher scores were recorded in the female sample, especially for the intrusive emotional experience (reliving negative feelings). Although this characteristic is typical of the period immediately following a traumatic event, its persistence over months seems to be a good predictor of long-term PTSD (45). This suggests that PTSD-related disorders resulting from COVID-19 could be a serious problem that needs addressing during the transition to post-pandemic conditions: interventions will be needed to support nurses to ensure a healthy workforce.

Differences in the prevalence of symptoms of PTSD could be explained by multiple factors, such as the specific temporal and epidemiological context in which the study was conducted, the characteristics of the organization (e.g., the presence of professional support for HCWs), and the context in which nurses are employed (e.g., type of COVID-19 work area) (16). In our study, nurses employed in the critical care area had a higher likelihood of a diagnosis of PTSD. This result is in line with data from the literature and previous pandemics (46–54). Nurses employed in critical care directly cared for COVID-19 patients and thus experienced patient deaths more frequently and had to make difficult decisions about the allocation of resources and equipment for the people in their care (41, 55–57). They were also exposed to greater health risks as a result of working with infected patients who required constant nursing care (58). In addition, female nurses, and nurses of both sexes with moderate-to-severe symptoms of GAD, reported more severe clinical predictors of PTSD. The literature supports these findings: in particular, a systematic review with a meta-analysis found a positive correlation between being female, anxiety, and PTSD during the pandemic (40), especially in nurses employed on the front line of care for COVID-19 patients (59). On the other hand, our model did not report associations for some predictors of PTSD described in the pandemic literature. Thus, marital status, being of young age, having worked continuously in COVID-19 units (first and second waves), and having been infected with COVID-19 were not found to be predictors of PTSD for the nurses examined, although they emerged as variables of interest in other studies (39, 60).

Some limitations of this study must be addressed. First, the selection of the sample was carried out by a snowball sampling procedure *via* social media using an online survey link. This has some limitations, as the method does not guarantee that the sample is representative of the larger population, especially due to the possibility that nurses with risk factors were more likely to participate and complete the survey. On the other hand, the selection method is easy to perform and allows the recruitment of a larger number of relevant individuals, and we therefore feel it is suitable for an exploratory analysis. Second, the survey used a self-reported questionnaire, which does not investigate the psychological status of nurses, especially for sensitive topics. However, to avoid bias, we adopted two validated questionnaires with reliable cut-offs that have been widely used in the study population. The above limitations have also been described in some of the pandemic literature and appear to be acceptable and understandable given the particularity of the pandemic context. In addition, the authors are aware that multiple other factors, such as a concurrent traumatic event experienced by HCWs, or pre-existing COVID-19 disorders, may have influenced our results. However, this summary allows us to obtain a current view of the emotional and psychological state of the nurses analyzed. Finally, the cross-sectional design of the study is a limitation, especially since it is a single measurement in a changing context (i.e., the pandemic); however, pending longitudinal analyses, these data can provide a useful overview of the health problem in question.

In conclusion, levels of PTSD symptoms and anxiety among nurses were high during the pandemic (27, 28) especially in the female sample (10, 61). These findings suggest that, as a primary prevention measure, screening for psychological problems among HCWs should be carefully conducted by healthcare organizations to protect the most vulnerable. Screening for anxiety disorders should be a priority; assessment can be conducted using commonly used validated instruments, such as the GAD-7 questionnaire. Indeed, assessment of anxiety disorders appears to be a reliable predictor for second-level screening of, for example, PTSD in employees with higher-than-average anxiety scores. This would enable organizations to identify the extent of the disorder early and to direct the at-risk population to targeted psychological support interventions. Routine assessment of nurses’ mental well-being is critical considering the results on reported PTDS symptoms, which seem to predict, especially for females, possible long-term sequelae (strong presence of intrusion symptoms). Finally, it seems necessary to implement gender policies in the post-pandemic period to address and modify support services specifically for female nurses, who have emerged as a vulnerable target population. The implementation of an early screening service for GAD and PTSD and the establishment of psychological programs for nurses (especially for those employed in critical care settings) seem to be mandatory actions that are required to promote organizational well-being and improve the quality of care. In addition, the scientific literature highlights that acute COVID-19 stress-related disorders and personal growth are linked and influenced by multiple factors. For this reason, it is necessary to develop support strategies that consider the resilience and emotions regulation skills of HCWs as an instrument for growth and psychological well-being. This strategy must be implemented at the organizational level with the purpose of promoting social support (62).

## Data availability statement

The raw data supporting the conclusions of this article will be made available by the authors, without undue reservation.

## Ethics statement

The studies involving human participants were reviewed and approved by the Institutional Ethics Board of the Policlinico Umberto I Teaching Hospital/Sapienza University of Rome (protocol number 0489/2021). The patients/participants provided their written informed consent to participate in this study.

## Author contributions

ER, PV, and AM contributed to the conception and design of the study. ER, DM, and GG performed data collection. ER, DM, and GG conducted the analyses and contributed to data curation. ER wrote the first draft of the manuscript. VI and AM wrote sections of the manuscript. VB, GM, CD, CM, PV, and AM critically revised the manuscript. All authors contributed to manuscript revision and read and approved the submitted version.

## Conflict of interest

The authors declare that the research was conducted in the absence of any commercial or financial relationships that could be construed as a potential conflict of interest.

## Publisher’s note

All claims expressed in this article are solely those of the authors and do not necessarily represent those of their affiliated organizations, or those of the publisher, the editors and the reviewers. Any product that may be evaluated in this article, or claim that may be made by its manufacturer, is not guaranteed or endorsed by the publisher.
